# Whole‐Genome Resequencing Reveals the Genetic Diversity and Adaptive Evolution of *Rhabdophis nuchalis*

**DOI:** 10.1002/ece3.74438

**Published:** 2026-09-28

**Authors:** Fei Wu, Jiu‐Yan Jiang, Ping‐Wei Liang, Ji Chen, Lin‐Ping Fan, Xia Zhou, Ji Wang, Ming‐Wen Duan, Yu‐Qi Cheng, Guang‐Xiang Zhu

**Affiliations:** ^1^ College of Life Science Sichuan Agricultural University Ya'an China; ^2^ School of Ecology and Environment Xizang University Lhasa China; ^3^ Chengdu Zoo Chengdu China

**Keywords:** adaptive selection, genetic differentiation, population genetic structure, *Rhabdophis nuchalis*

## Abstract

Snakes are valuable for studying adaptive evolution due to their morphological specialization, wide distribution, and strong adaptability. However, genomic studies on *Rhabdophis nuchalis* are limited. We resequenced 19 individuals from four regions to assess genetic diversity and adaptation. We obtained 39,226,324 high‐quality SNPs and 15,838,348 high‐quality INDELs. Genetic structure analysis showed Sichuan and Yunnan populations forming one cluster, and Hubei with Shaanxi another. Yunnan exhibited high nucleotide diversity and observed heterozygosity among the four sampled populations (Sichuan, Shaanxi, Hubei, and Yunnan), indicating substantial genetic diversity. Shaanxi showed low linkage disequilibrium, suggesting a larger effective population size, consistent with historical estimates. GO enrichment revealed differentiated genes related to reproduction and calcium‐dependent cell adhesion. KEGG identified only the cell adhesion molecules pathway. BLAST aligned these sequences with protocadherin β and γ subclusters. Overall, clear genetic clustering and differentiation exist among the four populations, with differentiation‐related genes involved in reproduction and cell adhesion, providing insights for genetic diversity conservation.

## Graphical abstract

*Rhabdophis nuchalis* populations from four regions were analyzed using genome resequencing. The results showed that the Sichuan and Yunnan populations formed one cluster, while the Hubei and Shaanxi populations formed another. The Yunnan population displayed the highest genetic diversity. Genes associated with population differentiation were mainly enriched in reproduction and calcium‐dependent cell adhesion functions.

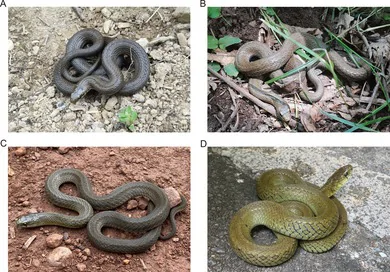

**Abbreviations**
BLAST: basic local alignment search tool
Gb: gigabase pair
Kb: kilobase pair
Mya: million years ago
*p*adj: adjusted *p* value
PI3K: phosphatidylinositol 3‐kinase
Rac: Rac family small GTPases

## 1 Background

Snakes represent a group of vertebrates with a high degree of morphological specialization (Zhao and Wang 2025). Current research indicates that snakes are widely distributed across all continents except for extreme polar environments (Jackson 2014). As efficient predators in ecosystems, snakes play a crucial role in maintaining ecological balance. Since diverging from their burrowing ancestors, snakes have undergone various adaptive evolutionary changes that distinguish them from most other squamates (e.g., lizards and amphisbaenians) (Peng et al. 2023). Certain snakes have also exhibited unique morphological and physiological characteristics during evolution. For example, rattlesnakes possess a rattle structure (Meik and Pires‐Dasilva 2009). Snakes of the families Viperidae and Elapidae produce highly toxic venom (Zhao 2006). Marine lineages within the subfamily Hydrophiinae have adapted to marine life (Sanders et al. 2008). Therefore, snakes serve as important subjects for studying genetic diversity and adaptive selection.

*Rhabdophis nuchalis* is a member of the genus *Rhabdophis*, belonging to the class Reptilia, order Squamata, suborder Serpentes, family Colubridae. It is named for the shallow groove formed by two parallel rows of dorsal scales on the midline of the neck (Zhao 2006). Throughout the taxonomic history, the nomenclature and classification of this species have undergone multiple revisions. It was originally described by Boulenger in 1891 as *Tropidonotus nuchalis* based on specimens collected from Yichang City, Hubei Province, China (Boulenger 1891). It was subsequently placed in the genus *Natrix sensu lato* (Parker 1925), and was later separated and established as *R. nuchalis* by Malnate in 1960 (Malnate 1960). In addition to taxonomic debates, its ecological traits also merit attention. Current research on *R. nuchalis* has largely focused on traditional fields, primarily involving morphology (Mori et al. 2016), phylogeny (Takeuchi et al. 2018), and biogeography (Zhu et al. 2022). In contrast, genomic studies remain scarce: to date, only a single‐sample genome assembly has been obtained (Duan et al. 2024), and there is a lack of in‐depth investigations incorporating multiple samples. To fill this gap, whole‐genome resequencing technology provides an important approach. Whole‐genome resequencing is one of the important applications of next‐generation sequencing (NGS), which provides a cost‐effective approach for such studies (DePristo et al. 2011). By aligning resequencing data to a reference genome, genetic variations can be identified, enabling investigations into species biodiversity and evolution. To date, only a few studies have applied resequencing technology to snakes (Tang et al. 2023; Li et al. 2018; Schield et al. 2020; Bernstein et al. 2025). Although previous studies have shown that *R. nuchalis* has a wide distribution range in China (Xie et al. 2019), the genetic structure and differentiation among its different geographical populations remain unresolved.

Therefore, this study aims to investigate the genetic diversity and adaptive evolution characteristics of *R. nuchalis* using whole‐genome resequencing. A total of 19 individuals of *R. nuchalis* collected from four different regions in China—Sichuan (SC), Yunnan (YN), Shaanxi (SX), and Hubei (HB) (Figure 1)—were subjected to whole‐genome resequencing, followed by analyses of genetic diversity and population structure. Combined with genome‐wide selective signal analysis, key pathways exhibiting significant genetic differentiation between populations from different regions, as well as homologous gene fragments under selection, were preliminarily identified. This study provides a theoretical basis and data support for the formulation of conservation strategies for *R. nuchalis* and the maintenance of regional biodiversity, and serves as a research case for genomic studies on non‐model organisms.

**FIGURE 1:**
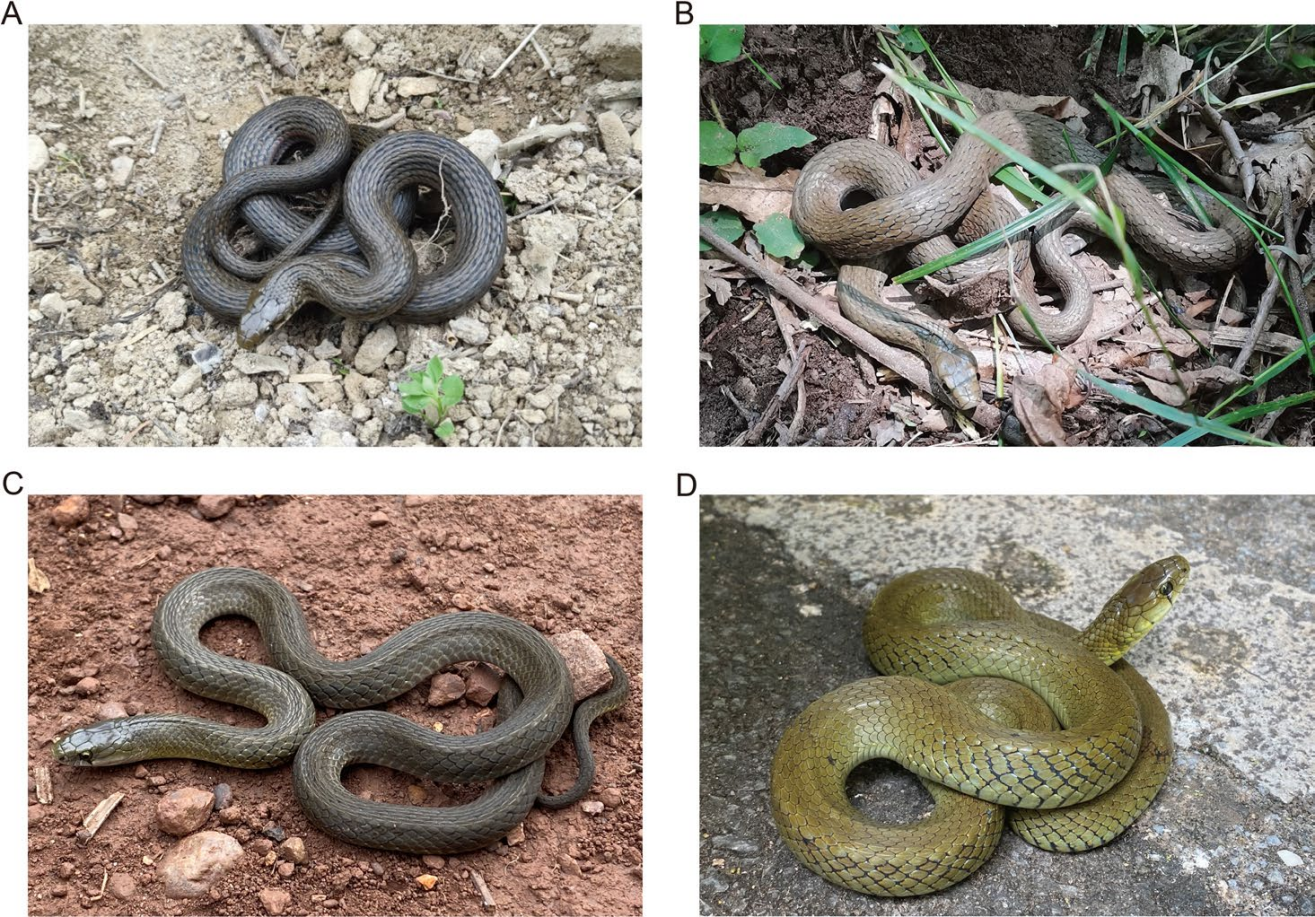
Photographs of *Rhabdophis nuchalis* from four regions: (A) Hubei, (B) Shaanxi, (C) Sichuan, (D) Yunnan. Photographs (A–C) were taken by Guang‐Xiang Zhu, and photograph (D) was taken by Jing‐Song Shi. The photographer of photograph (D) is not an author of this study.

## 2 Materials and Methods

### 2.1 Sample Collection

We collected three specimens from Shennongjia in Hubei Province in May 2023, and between June and August 2024, we obtained eight, five, and three specimens from Ankang City in Shaanxi Province, Panzhihua City in Sichuan Province, and Dali Bai Autonomous Prefecture in Yunnan Province, respectively. All specimens were randomly collected in the field from mountainous forests at elevations ranging from 1200 to 2750 m above sea level (https://reptile‐database.reptarium.cz/) (Table S1).

The collected specimens were securely placed in transparent plastic bottles and transported to the Zoology Laboratory of Sichuan Agricultural University. Prior to tissue collection, all individuals were euthanized by rapid decapitation. Liver tissue samples were then collected, placed into sterilized 2 mL Eppendorf tubes, immediately frozen in liquid nitrogen, and subsequently stored at −80°C. All experimental procedures were approved by the Institutional Animal Care and Welfare Committee of Sichuan Agricultural University (Approval No. 20220125). The collected liver tissue samples were subsequently sent to Shanghai Personal Biotechnology Co. Ltd. for resequencing.

### 2.2 DNA Extraction and Whole‐Genome Resequencing

An appropriate amount of frozen sample was placed into a centrifuge tube and immediately immersed in liquid nitrogen. The sample was ground using a tissue homogenizer. After removal from liquid nitrogen, 700 μL of 2× CTAB extraction buffer was added, mixed thoroughly, and incubated in a 65°C water bath with intermittent inversion mixing. The supernatant was transferred to a new tube, and 2 μL of RNase was added for 5 min at room temperature, followed by a second extraction with chloroform/isoamyl alcohol. The supernatant was transferred to a new tube, and an equal volume of isopropanol or two volumes of absolute ethanol was added to precipitate the DNA. After centrifugation at 12,000 rpm for 10 min, the supernatant was discarded. The transparent DNA pellet was washed once with 80% ethanol, air‐dried, and dissolved in 30–50 μL of nuclease‐free water. Total genomic DNA was extracted from liver tissue samples. The quality of the extracted DNA was assessed by 0.8% agarose gel electrophoresis, and the DNA concentration was quantified using a UV spectrophotometer.

Qualified DNA samples were used for library construction with an insert size of 400 bp. The constructed libraries, after passing quality control, were subjected to paired‐end sequencing (2 × 150 bp) on the BGISEQ DNBSEQ‐T7 platform, with a target sequencing depth of 10× .

The raw data after sequencing were saved in paired‐end FASTQ format. To ensure the quality of subsequent bioinformatics analysis, the raw data were further filtered to generate high‐quality data. The raw data were filtered using fastp (v0.23.1) (Chen et al. 2018) with a sliding window approach. The filtering criteria were as follows: (1) removal of adapter contamination by trimming adapter sequences from the 3′ ends; (2) quality filtering using a sliding window method with a window size of 5 bp and a step size of 1 bp. At each step, the window moved forward by one base, and the average *Q*‐score of the five bases within the window was calculated. If the *Q*‐score of the last base was ≤ 20, only the bases preceding that position were retained; if the average *Q*‐score of the window was ≤ 20, only the bases up to and including the second‐to‐last base of the window were retained; (3) if the length of either read in a paired‐end pair was ≤ 50 bp, the entire paired‐end reads were discarded.

### 2.3 Sequence Alignment

The quality‐controlled data were aligned to the published reference genome sequence of *R. nuchalis* (GenBank accession No. GCA_039707465.1) (Duan et al. 2024) using Burrows‐Wheeler Aligner (BWA) (Li 2013) (v0.7.19‐r1273) for sequence mapping. Using Samtools (v1.20) (Li et al. 2009), we calculated genome coverage for each sample and performed format conversion and sorting of BAM files. Picard (http://broadinstitute.github.io/picard) was used to mark PCR duplicates.

### 2.4 Variant Calling

Variant calling was performed using the HaplotypeCaller module of GATK (v4.2.6) (McKenna et al. 2010). The CombineGVCFs module was used to merge the GVCF files from all samples. Joint genotyping was then performed using the GenotypeGVCFs module to obtain a raw VCF file containing variant information for all samples. Finally, the SelectVariants module was used to screen for single nucleotide polymorphisms (SNPs) and insertions and deletions (INDELs). After obtaining the raw SNPs, filtering was performed based on the following criteria: QD (Quality by Depth) < 2.0, MQ (Mapping Quality) < 40.0, FS (Fisher Strand) > 60.0, SOR (Strand Odds Ratio) > 3.0, MQRankSum < −12.5, and ReadPosRankSum < −8.0. To ensure accuracy of the results, SNPs were further retained with the following criteria: QUAL > 20, minor allele frequency (MAF) > 5%, and missing rate < 10%. Raw INDELs were filtered by retaining variants with QUAL > 20 and MAF > 5%, and excluding variants with QD < 2.0, FS > 200.0, SOR > 10.0, or ReadPosRankSum < −20.0, and high‐quality INDELs were retained.

### 2.5 Population Genetic Structure Analysis

The filtered SNPs were further filtered prior to principal component analysis (PCA) using PLINK (Purcell et al. 2007): individuals with a SNP missing rate ≤ 10% were retained, loci meeting the Hardy–Weinberg equilibrium (HWE) test with *p* ≥ 0.0001 were retained, and linkage disequilibrium (LD) pruning was performed (‐‐indep‐pairwise 50 5 0.5). PCA was performed based on the degree of genomic SNP variation among the filtered SNPs to cluster individuals into different subgroups. A phylogenetic tree was constructed based on SNPs using FastTree (Price et al. 2010) with the following parameters: ‐nt ‐gtr ‐gamma ‐boot 1000 ‐cat 20. Population structure analysis of *R. nuchalis* populations from the four regions was performed using Admixture (Alexander et al. 2009). First, PLINK (Purcell et al. 2007) was used for LD pruning (‐‐indep‐pairwise 50 5 0.5) and format conversion, and the pruned SNPs were used for subsequent analysis. Clustering was performed with a predefined number of ancestral populations (*K* value) ranging from 1 to 8, and the *K* value with the minimum cross‐validation error was selected as the optimal number of populations. To mitigate the influence of stochastic initialization, we performed 10 independent runs for each *K* value, each with a different random seed generated by the system. The run showing the clearest clustering patterns and the smoothest CV error trend was selected as the representative result for visualization and downstream analyses, as the CV error curves were highly consistent across replicates, indicating stable convergence.

### 2.6 Population Genetic Diversity Analysis

VCFtools (Danecek et al. 2011) was used to scan the VCF file and calculate genomic nucleotide diversity (*π*) for each population. The VCF file was scanned using a window size of 20 Kb with a step size of 10 Kb across the genome. Meanwhile, the sliding window method was used to calculate *F*st values among different *R. nuchalis* populations. PLINK (Purcell et al. 2007) was used to calculate observed heterozygosity (Ho) and expected heterozygosity (He). To assess linkage disequilibrium (LD) patterns, PopLDdecay (Zhang et al. 2018) was used to calculate the squared correlation coefficient of allele frequencies (*r* ^2^).

### 2.7 Population Historical Dynamics Analysis

Effective population size (Ne) was inferred using SMC++ (Terhorst et al. 2017). First, the vcf2smc tool was used to convert the quality‐controlled VCF format variant data into an input format compatible with SMC++. The generation time of *Rhabdophis* was estimated as three years based on age at sexual maturity data from other species within the same family (Colubridae), specifically three to four years for *Boiga irregularis* (Savidge et al. 2007) and three years for *Coronella austriaca* (Goddard 1981). Given the absence of species‐specific mutation rate reports for the genus *Rhabdophis* or its closely related colubrid taxa, we adopted the mutation rate of *Hydrophis curtus* (Peng et al. 2020), namely 4.71 × 10^−9^ per site per year, which represents one of the few reliable mutation rate estimates available for the suborder Serpentes.

### 2.8 Selective Signal Scanning and Functional Enrichment Analysis

VCFtools (Danecek et al. 2011) was used to perform selective signal analysis between pairwise populations. Using nucleotide diversity (*θπ*) and the genetic differentiation coefficient (*F*st) as indicators, windows that simultaneously satisfied both criteria (*F*st in the top 5% and *θπ* in the bottom 5%) were selected as candidate regions for adaptive evolution. Functional enrichment analysis of genes located within the identified selected regions was performed using TBtools (Chen et al. 2023), including Gene Ontology (GO) and Kyoto Encyclopedia of Genes and Genomes (KEGG) enrichment analyses.

To distinguish genuine signals of positive selection from genomic regions potentially influenced by demographic history (e.g., population bottlenecks or founder effects), we performed a neutrality validation test based on Tajima's *D* (Tajima 1989). We compared the distribution of Tajima's *D* between candidate windows and matched control windows.

For each pairwise population comparison, we independently constructed a matched set of control windows. For each candidate window (20 Kb), we first calculated GC content using Bedtools (Quinlan and Hall 2010). For each candidate window, we identified the 50 non‐candidate windows with the most similar GC content across the genome and randomly selected five of them as controls. All candidate windows were merged into a set of candidate regions, and all control windows were merged into a set of control regions. This matching strategy ensured that candidate and control windows were comparable in their basic genomic properties, allowing any observed differences in Tajima's *D* to be attributed to selection rather than to intrinsic sequence characteristics.

Tajima's *D* (Tajima 1989) was calculated for each candidate and control window using VCFtools (Danecek et al. 2011) with a 20 Kb sliding window (parameter ‐‐TajimaD 20000) within the candidate and control region sets, applied to the individual VCF files of each population (HB, SC, SX, and YN). Only biallelic SNPs were retained for analysis, and windows containing fewer than three SNPs were excluded.

For each population within each pairwise comparison, we compared the distribution of Tajima's *D* values between candidate windows and their matched control windows. We performed a one‐sided Wilcoxon rank‐sum test, with the alternative hypothesis that the median Tajima's *D* in candidate windows is significantly lower than that in control windows. The Wilcoxon rank‐sum test was chosen for its non‐parametric nature, as Tajima's *D* values did not always conform to normality. All statistical analyses were conducted in R v4.3.3 (R Core Team 2024), with *p* < 0.05 considered statistically significant.

## 3 Results

### 3.1 Whole‐Genome Resequencing and SNP Calling

In this study, whole‐genome resequencing was performed on 19 *R. nuchalis* samples, generating 277.2 Gb of raw data. After quality control, 268.1 Gb of high‐quality data were obtained, with an average Q20 of 98.75% and an average Q30 of 96.51%. The high‐quality data were successfully aligned to the reference genome, achieving an average coverage depth of 11.5× and an average mapping rate of 99.71% (Table S2). Variant calling using GATK identified a total of 84,612,445 single nucleotide polymorphisms (SNPs) and 16,002,200 insertions and deletions (INDELs). After quality control, 39,226,324 high‐quality SNPs and 15,838,348 high‐quality INDELs were obtained.

### 3.2 Population Genetic Structure Analysis

Principal component analysis (PCA) showed that the first principal component (PC1) explained 28.5% of the total variance and effectively distinguished two major groups: the SC and YN populations clustered together, clearly separating from the HB and SX populations. The second principal component (PC2) explained 15.19% of the total variance and further revealed differentiation between the SC and YN populations, while the HB and SX populations remained tightly clustered without showing genetic differentiation (Figure 2A). These results suggest that strong genetic differentiation exists between the populations from the SC‐YN region and the HB‐SX region.

**FIGURE 2:**
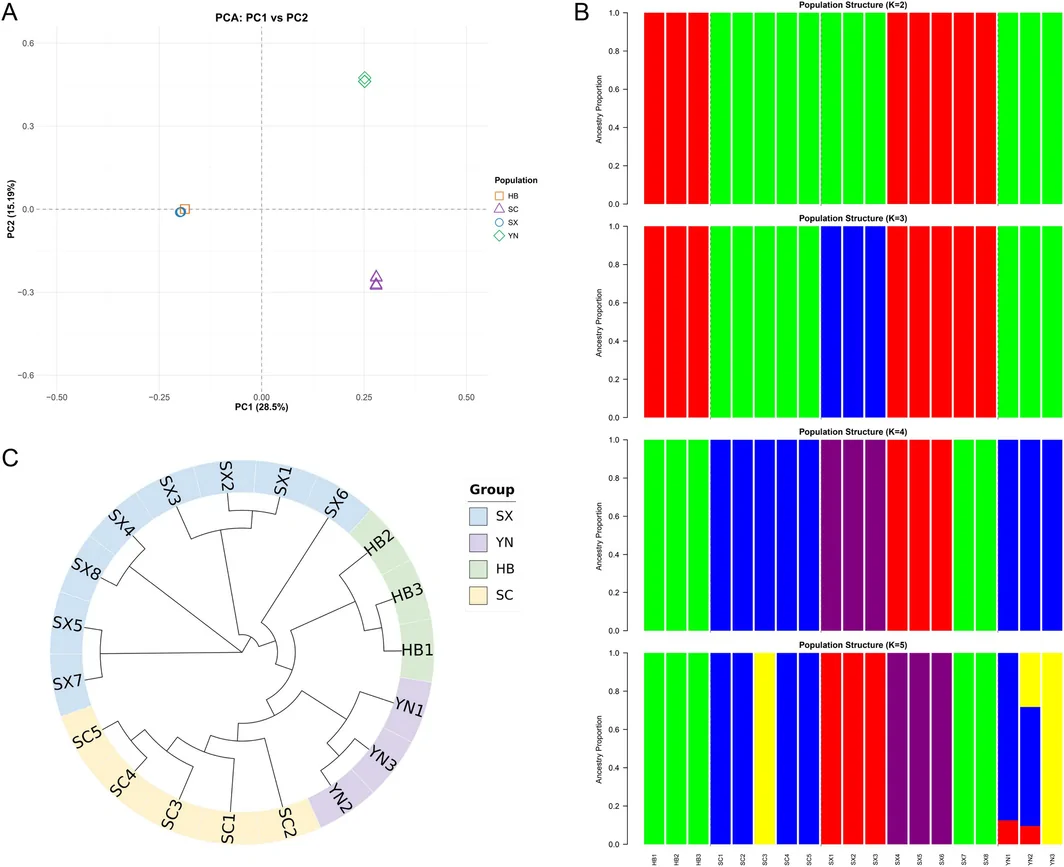
Genetic structure analysis diagram. (A) Principal component analysis of four populations. (B) Genetic structure clustering plot for ancestral population numbers (*K* = 2–5). (C) Phylogenetic tree based on SNPs data.

Admixture analysis of population structure showed that when the number of ancestral components (*K*) was 2, the four geographical populations of *R. nuchalis* could be divided into two major genetic groups: all individuals from the YN and SC populations, together with some individuals from the SX population, shared the same genetic component and formed one cluster; the remaining SX individuals formed the other cluster together with the HB individuals. When *K* = 3, the SX population still exhibited two subclusters, but showed genetic separation from the SC and YN populations. When *K* = 4, further subdivision of genetic components was observed within the SX population, which exhibited three distinct subclusters (Figure 2B). This suggests the occurrence of historical or contemporary migration events among populations from different regions. Cross‐validation error analysis indicated that the model achieved the minimum cross‐validation (CV) error when *K* = 2, indicating that *K* = 2 is the best‐supported *K* among the tested models (Table S3). Phylogenetic tree analysis based on SNPs showed that the HB, YN, and SC populations each formed distinct clusters, with YN and SC clustering together as a major lineage, while the SX population did not form a monophyletic clade, suggesting potential internal differentiation within this population (Figure 2C), which was consistent with the results of the Admixture analysis.

### 3.3 Population Genetic Diversity Analysis

Nucleotide diversity (*θπ*) analysis showed that the YN population exhibited the highest nucleotide diversity, while the SC population had the lowest genetic diversity (Figure 3A). The observed heterozygosity was lower than the expected heterozygosity in all populations, with the YN population showing the highest observed heterozygosity and the HB population the lowest (Table 1). This suggests that the YN population possesses a high level of genetic diversity. All populations exhibited heterozygote deficiency, as indicated by Ho < He.

**FIGURE 3:**
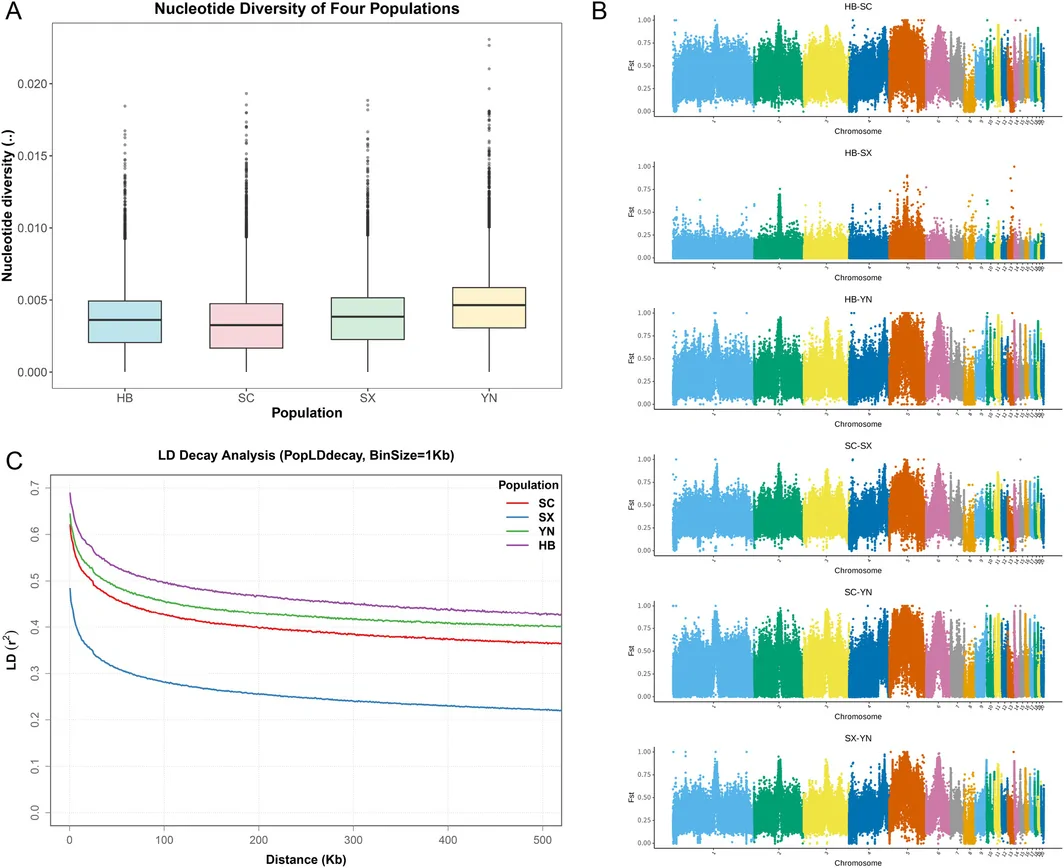
Genetic diversity analysis diagram. (A) Boxplot of nucleotide diversity among four populations. (B) Pairwise *F*st between populations. (C) Linkage disequilibrium analysis results of four local populations.

**TABLE 1:** Analysis results of nucleotide diversity and heterozygosity for four local populations.

Population name	Average nucleotide diversity (*π*)	Observed heterozygosity (Ho)	Expected heterozygosity (He)
HB	0.00352405	0.0282014	0.316722
SX	0.00372179	0.0356205	0.110932
SC	0.00330973	0.0315714	0.184179
YN	0.00437851	0.0379944	0.311125

Linkage disequilibrium (LD) decay analysis showed that the SX population exhibited the fastest LD decay rate, while the YN, HB, and SC populations showed relatively slower LD decay rates. At 100 Kb, the LD level (*r* ^2^) of the SX population was lower than that of the other three populations. The LD levels of the YN, HB, and SC populations were relatively similar to each other and consistently higher than those of the SX population (Figure 3C).

Calculation of the fixation index (*F*st) between pairwise populations showed that the differentiation between the HB and SX populations was relatively low compared to other population pairs (Figure 3B), suggesting that the genetic differentiation between the HB and SX populations is low and that there may be strong gene flow between them.

### 3.4 Population Historical Dynamics Analysis

Coalescent analysis of historical effective population size (Ne) for the four *R. nuchalis* populations was performed. The results showed that, overall, the historical effective population sizes of all four populations exhibited a declining trend, with each population showing a slight increase in the early stage. Among them, after an initial slight increase, the effective population sizes of the HB, SC, and YN populations experienced a sharp decline. Although minor fluctuations occurred during this process, the overall trend remained continuously declining. In contrast, the SX population showed a dynamic pattern characterized by an initial fluctuating decline, followed by a rapid recovery, and then another decline gradually leveling off (Figure 4).

**FIGURE 4:**
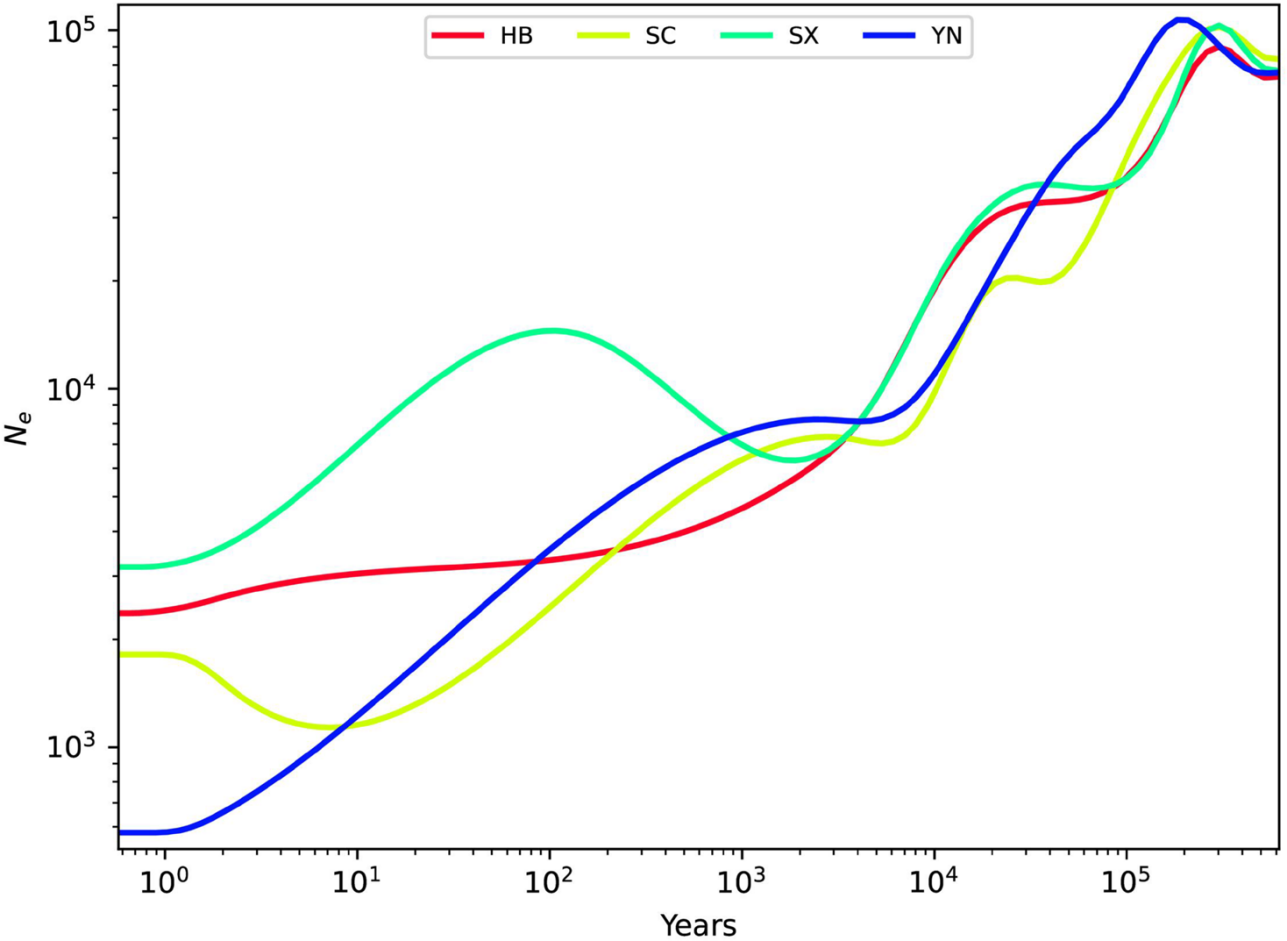
Historical effective population sizes of the four populations.

### 3.5 Selective Sweep Analysis

Candidate regions were defined as genomic windows with *F*st values in the top 5% and *θπ* values in the bottom 5%. Selective sweep analyses were conducted between each pair of populations, and genes within the candidate regions for each population pair were screened. The results are shown in Table S4. These genes were then subjected to GO and KEGG enrichment analyses. In GO enrichment analysis, no significant enrichment (*p*adj < 0.05) was observed for the HB versus SX population pair. For the remaining population pairs, the genes under selection between HB and SC were primarily enriched in multi‐organism reproductive process (GO:0044703) and developmental process (GO:0032502) (Figure 5A). The only significantly enriched GO term between the SX and YN populations was oogenesis (GO:0048477) (Figure 5B). Significantly differentiated genes between the HB and YN populations were significantly enriched in calcium‐dependent cell–cell adhesion (GO:0016339) (Figure 5C). Genes between the SC and SX populations were significantly enriched in germ cell development (GO:0007281), multicellular organismal reproductive process (GO:0048609), and gamete generation (GO:0007276) (Figure 5D). Genes between the SC and YN populations were significantly enriched in calcium‐dependent cell–cell adhesion (GO:0016339) (Figure 5E). In KEGG analysis, no significant enrichment (*p*adj < 0.05) was detected for the HB versus SX, SX versus YN, and SC versus SX population pairs. For the HB versus YN, HB versus SC, and SC versus YN population pairs, all KEGG enrichment analyses showed significant enrichment in the cell adhesion molecules (map04515) pathway (Figure 6).

**FIGURE 5:**
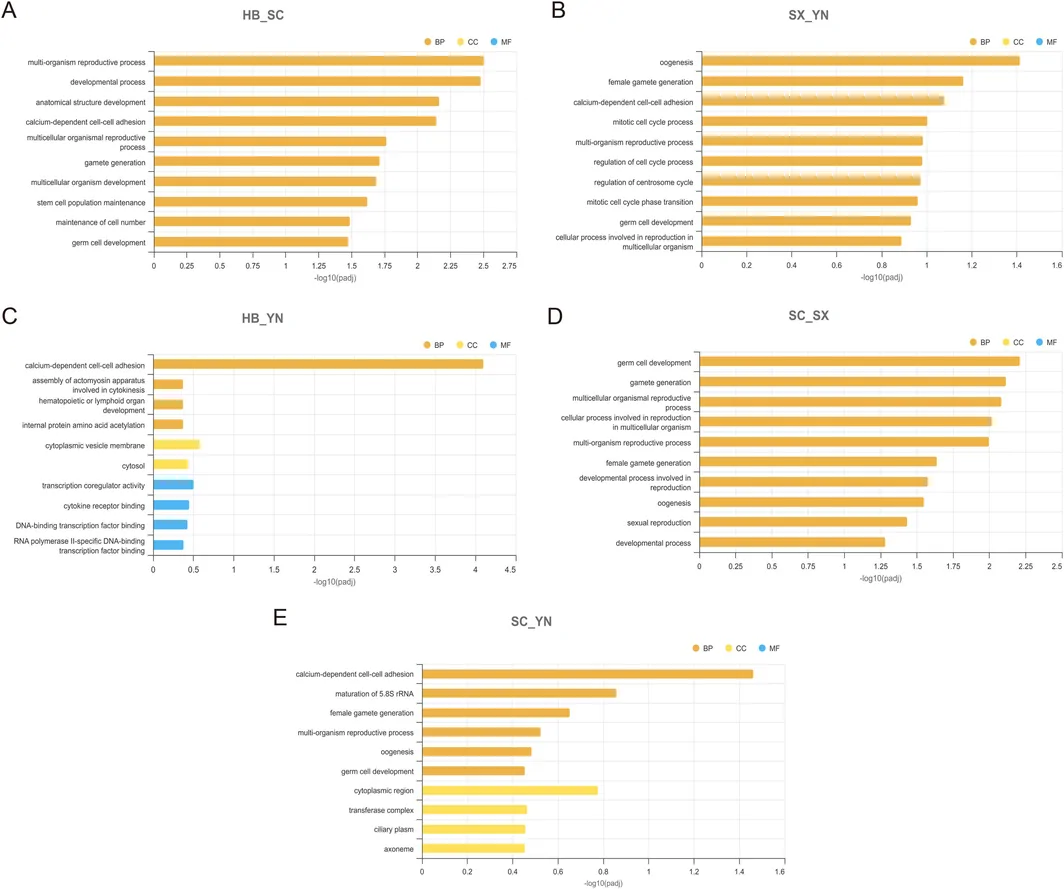
GO functional enrichment analysis of significantly differentiated genes among different populations of *Rhabdophis nuchalis* . (A) GO enrichment analysis of significantly differentiated genes between HB and SC populations. (B) GO enrichment analysis of significantly differentiated genes between SX and YN populations. (C) GO enrichment analysis of significantly differentiated genes between HB and YN populations. (D) GO enrichment analysis of significantly differentiated genes between SC and SX populations. (E) GO enrichment analysis of significantly differentiated genes between SC and YN populations.

**FIGURE 6:**
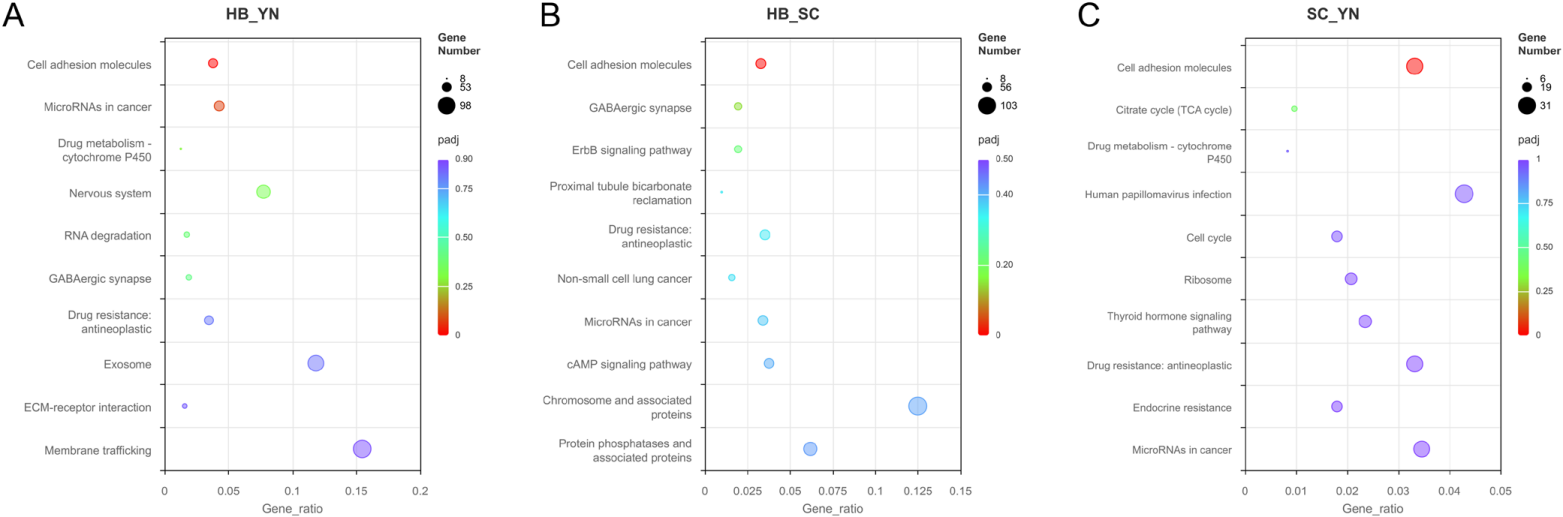
Results of KEGG enrichment analysis of significantly differentiated genes in different populations of *Rhabdophis nuchalis* . (A) KEGG enrichment analysis of significantly differentiated genes between HB and YN populations. (B) KEGG enrichment analysis of significantly differentiated genes between HB and SC populations. (C) KEGG enrichment analysis of significantly differentiated genes between SC and YN populations.

Integrating the GO/KEGG enrichment analyses with the Tajima's *D* validation results (Table S5), we observed distinct patterns of functional enrichment and selection signals across different population comparisons. In HB‐inclusive comparisons, although significant enrichments were detected in GO and KEGG analyses, Tajima's *D* validation revealed no significant selective sweep signals in the HB population itself, suggesting that the differentiation signals in HB‐related comparisons may primarily derive from selective events in the other three populations, while the HB population may have formed a background of differentiation due to demographic history or genetic drift. In non‐HB‐inclusive comparisons, Tajima's *D* validation confirmed that SC, SX, and YN populations all exhibited significant selection signals. Taken together, these results suggest that SC, SX, and YN populations may have played a more important role in the differentiation processes observed in this study.

## 4 Discussion

The first principal component (PC1), as the main component explaining genetic variation in *R. nuchalis* populations, effectively distinguished the two major geographic genetic groups, with the SC and YN populations clearly separated from the HB and SX populations, indicating genetic differentiation between populations from these two geographic regions. The geographic isolation hypothesis is a mainstream theory explaining disjunct distribution in snakes (Esquerré et al. 2020; Weinell et al. 2020). Zhu et al. found that the Sichuan Basin forms a geographic barrier hindering species migration (Zhu et al. 2022), which may impede gene flow between the SC‐YN and HB‐SX geographic regions, allowing genetic differences to gradually accumulate during long‐term evolutionary processes.

Admixture analysis of population structure showed that when the optimal number of ancestral components was 2 (*K* = 2), the four geographical populations of *R. nuchalis* could be clearly divided into two genetic clusters. All individuals from the YN and SC populations, together with some individuals from the SX population, shared the same ancestral genetic component and formed one cluster, while the remaining SX individuals formed the other cluster together with the HB individuals. This genetic distribution pattern, which is not strictly clustered according to geographic isolation, suggests that gene flow or historical dispersal events may exist among *R. nuchalis* populations in the study area, rather than strict geographic differentiation. In 2022, Zhu et al. found that *R. nuchalis* originated in the Hengduan Mountains and expanded to the Qinling‐Daba Mountains, and that the *R. nuchalis* populations from Sichuan, Shaanxi, and Hubei diverged into two clades at approximately 1.54 Mya, with individuals from Sichuan and Hubei forming clades with some individuals from Shaanxi, respectively (Zhu et al. 2022). This pattern, in which individuals from Shaanxi were assigned to different clusters, is similar to our Admixture results. Some scholars have suggested that the high mountain valley regions of western Yunnan, the Nanling Mountains spanning Guangdong, Guangxi, Hunan, and Jiangxi, and the border areas of Hunan, Sichuan, and Hubei were less affected by Quaternary glaciations and served as important refugia (Wang and Liu 1994). Klicka and Zink proposed that glacial refugia could promote intraspecific subspecific differentiation (Klicka and Zink 1997). The observed genetic differentiation between the SC‐YN and HB‐SX groups may be linked to isolation in distinct refugia since the last glacial period, while the admixed ancestry detected in the SX population likely reflects gene flow during subsequent dispersal or secondary contact. Nevertheless, this scenario remains speculative and highlights the need for demographic model comparison or spatial phylogeographic testing.

Nucleotide diversity (*θπ*) and heterozygosity analyses collectively revealed differences in genetic diversity among different geographical populations. Among them, the YN population exhibited the highest nucleotide diversity, which may be attributed to the rich species diversity and ecosystem types in Yunnan (Yang et al. 2004), suggesting that this population may have maintained stable accumulation of genetic variation over a long period. The observed heterozygosity (Ho) was significantly lower than the expected heterozygosity (He) in all populations, indicating a possible excess of homozygotes, which reflects the Wahlund effect (Sinnock 1975) (i.e., the presence of undifferentiated subpopulations within each population) or may be due to a certain degree of inbreeding within the populations, but the specific contributing factors need to be distinguished through further analysis. Although the YN population had the highest observed heterozygosity among all populations, its Ho was still lower than He, indicating that even populations with relatively high genetic diversity may be affected by other factors.

Linkage disequilibrium analysis showed that the SX population exhibited the fastest LD decay rate, and its LD level (*r* ^2^) was significantly lower than that of the other three populations, indicating that the SX population may have a higher recombination rate or a larger effective population size. This is consistent with our historical effective population size estimates, which showed that the SX population maintained a relatively large effective population size among the four populations in recent history.

Historical effective population size analysis showed that the HB, SC, and SX populations experienced minor fluctuations between 0.1 and 0.01 Ma, with brief recoveries or stabilization followed by rapid declines. This may be related to the most recent major glaciation event in China, the Dali Glaciation (0.07–0.01 Mya) (Shi and Wang 1979). In contrast, the YN population exhibited a sharp decline in effective population size, which may be attributed to the lack of suitable refugia or intense intraspecific competition within refugia. The effective population size of the SX population showed a rapid increase around 0.001 Mya, which may be associated with the Taibai Glaciation (0.01862–0.00073 Mya) (Rost 1994; Zhang et al. 2014).

In GO enrichment analysis, no significantly enriched GO terms (*p*adj < 0.05) were detected for the HB versus SX population pair, whereas all other population pairs exhibited distinct differences in enriched functional categories. This result may be related to the relatively low genetic differentiation between HB and SX, although the influence of other factors—such as gene flow or convergent selection—cannot be excluded and would require further investigation. Combined with the Tajima's *D* validation results, the differentiation signals described above are primarily localized to the SX, SC, and YN populations. Reproductive‐related functions (reproductive process, oogenesis, germ cell development, gamete generation) were repeatedly and significantly enriched across multiple population pairs, suggesting that differentiation in reproductive‐related genes is one of the core drivers of intraspecific population differentiation in this species. Studies have shown that in populations adapting to new environments, reproductive isolation can evolve rapidly (Hendry et al. 2000). The significant differentiation of reproductive pathways among populations in this study is consistent with this conclusion, indicating that such differentiation may facilitate intraspecific genetic divergence. For the YN versus HB and YN versus SC population pairs, only calcium‐dependent cell–cell adhesion was significantly enriched. Cadherin superfamily members may play a role in this process. A study has shown that N‐cadherin primarily mediates cell–cell interactions and can interact with various proteins, affecting their localization and function, thereby establishing molecular polarity (Herrera et al. 2021). N‐cadherin can interact with actin to regulate cell contraction and movement, and can also interact with signaling molecules such as PI3K and Rac, influencing their aggregation and activity at the leading edge of cells, thereby regulating directional cell migration (Choi et al. 2021; Nguyen et al. 2019; Shellard and Mayor 2021; Wang et al. 2022). To initiate directional movement, cells must establish polarity (Lavrsen et al. 2023). Directional migration of cells in vivo is crucial for tissue remodeling and organismal survival (SenGupta et al. 2021; Yamada and Sixt 2019). The YN population is distributed in Yunnan, a region characterized by high biodiversity. The enrichment of this pathway suggests that it may play a role in local environmental adaptation in Yunnan; however, the underlying mechanisms require further functional validation.

In KEGG analysis, no significantly enriched pathways (*p*adj < 0.05) were detected for the HB versus SX, SX versus YN, and SC versus SX population pairs. For the HB versus YN, HB versus SC, and SC versus YN population pairs, KEGG analysis only identified the cell adhesion molecules pathway. Tajima's *D* validation revealed that the differentiation signals were primarily localized to the SC and YN populations. Dynamic cell adhesion plays a crucial role in cell migration (Yamada and Sixt 2019; Doyle et al. 2015; Case and Waterman 2015). BLAST alignment of gene sequences enriched in the cell adhesion molecules pathway revealed that these sequences were highly homologous to fragments of the *β* and *γ* subclusters of the protocadherin gene family. Protocadherin genes are primarily involved in neuronal connectivity and intercellular communication in vertebrates, serving as key gene families regulating nervous system development (Takeichi 2007; Lv et al. 2022; Jia and Wu 2020). Therefore, differentiation in these fragments may suggest that populations from different geographical regions have undergone adaptive differentiation in nervous system‐related genes in response to local environmental pressures. Notably, the *γ* subcluster of protocadherins, which shares homology with the gene sequences under selection, exhibits complex alternative splicing characteristics. Differentiation in these fragments may lead to differences in splicing patterns or isoform expression among different geographical populations during adaptive differentiation.

Based on the genetic characteristics described above, we suggest that the Yunnan population, which exhibits the highest genetic diversity and is located in a biodiversity hotspot, be prioritized for conservation. The Shaanxi population, by contrast, shows mixed ancestry among individuals, suggesting that this region may represent an admixture zone between different genetic groups; thus, its genetic composition warrants continued monitoring. Furthermore, given the genetic differentiation among the major geographic populations, we recommend that they be managed as independent management units, with priority given to genetic diversity monitoring in the Yunnan population.

## 5 Limitations

It should be noted that the results of this study may be limited to some extent by sample size. The relatively small and unevenly distributed sample size may have some influence on allele frequency estimation and the assessment of genetic diversity levels. Additionally, due to the lack of species‐specific mutation rate parameters for the genus *Rhabdophis*, we used the mutation rate of *Hydrophis curtus* for population history inference, which may introduce certain uncertainties in the accurate estimation of historical dynamics. Therefore, we tend to interpret the above results with caution. Future studies should incorporate larger population samples and more precise species‐specific parameters to further validate and deepen our understanding of the adaptive evolutionary mechanisms in *Rhabdophis nuchalis* .

## 6 Conclusion

This study clarifies the genetic structure and adaptive differentiation patterns of *R. nuchalis* populations from different geographical regions. The Yunnan population exhibits high genetic diversity, while the Shaanxi population maintains a large historical effective population size, providing important reference value for the conservation of genetic resources in this species. Reproductive processes and cell adhesion pathways may play critical roles in species genetic differentiation by regulating reproduction and cell communication functions. These findings deepen our understanding of adaptive selection in *R. nuchalis* and provide a scientific basis for formulating population management strategies centered on genetic resource conservation, while also offering new insights into microevolution in reptiles.

## Author Contributions

**Fei Wu:** conceptualization (lead), data curation (lead), formal analysis (lead), investigation (equal), methodology (lead), writing – original draft (lead), writing – review and editing (lead). **Jiu‐Yan Jiang:** investigation (equal), methodology (equal), writing – review and editing (equal). **Ping‐Wei Liang:** investigation (equal), methodology (equal), writing – review and editing (equal). **Ji Chen:** investigation (equal), methodology (equal), writing – review and editing (equal). **Lin‐Ping Fan:** investigation (equal), methodology (equal), writing – review and editing (equal). **Xia Zhou:** investigation (equal), methodology (equal), writing – review and editing (equal). **Ji Wang:** investigation (equal), methodology (equal), writing – review and editing (equal). **Ming‐Wen Duan:** investigation (equal), methodology (equal), writing – review and editing (equal). **Yu‐Qi Cheng:** investigation (equal), methodology (equal), writing – review and editing (equal). **Guang‐Xiang Zhu:** conceptualization (equal), funding acquisition (lead), resources (lead), supervision (lead), writing – original draft (supporting), writing – review and editing (equal).

## Funding

This work was supported by the National Natural Science Foundation of China (NSFC 32270477).

## Ethics Statement

This study was approved by the Institutional Animal Care and Welfare Committee of Sichuan Agricultural University (Approval No. 20220125) and followed the relevant national regulations on animal welfare and ethics.

## Consent

The authors have nothing to report.

## Conflicts of Interest

The authors declare no conflicts of interest.

## Supporting information


**Table S1:** *Rhabdophis nuchalis* sample collection information.

**Table S2:** Sample data information sheet.

**Table S3:** Cross‐validation errors for *K* = 1–8.

**Table S4:** Number of windows and genes identified by selective sweep analysis in the population.

**Table S5:** Comparison of Tajima's *D* between candidate windows and matched control windows.

## Data Availability

The dataset(s) supporting the conclusions of this article is(are) available in the NCBI Sequence Read Archive (SRA) repository, PRJNA1451851. https://www.ncbi.nlm.nih.gov/bioproject/PRJNA1451851.
