# Supplementary material for: Whole‐Genome Resequencing Reveals the Genetic Diversity and Adaptive Evolution of Rhabdophis nuchalis
Source: Ecol Evol. 2026 Sep 28;16(10):e74438. doi: 10.1002/ece3.74438 (PMC13619356; doi:10.1002/ece3.74438)
Supplement: Supplementary file 1 [file ECE3-16-e74438-s001.pdf]

Supplementary Table 3. Cross-validation errors for K=1-8

K value	CV error
K=1	0.81415
K=2	0.80418
K=3	1.07842
K=4	1.21697
K=5	1.51250
K=6	1.64261
K=7	1.87599
K=8	1.58442
