# Supplementary material for: Whole‐Genome Resequencing Reveals the Genetic Diversity and Adaptive Evolution of Rhabdophis nuchalis
Source: Ecol Evol. 2026 Sep 28;16(10):e74438. doi: 10.1002/ece3.74438 (PMC13619356; doi:10.1002/ece3.74438)
Supplement: Supplementary file 2 [file ECE3-16-e74438-s002.pdf]

Supplementary Table 5. Comparison of Tajima's D between candidate windows and matched control windows.

pair	population	median_candidate	median_control	p_value	significant
HB_SC	HB	0.684335	0.289548	1.000	No
HB_SC	SC	0.180681	0.547707	<0.001	Yes
HB_SX	HB	0.685751	0.292167	1.000	No
HB_SX	SX	-0.257375	0.4955725	<0.001	Yes
HB_YN	HB	0.667483	0.29029	1.000	No
HB_YN	YN	0.406628	0.585161	<0.001	Yes
SC_SX	SC	0.192083	0.547621	<0.001	Yes
SC_SX	SX	-0.2616365	0.513103	<0.001	Yes
SC_YN	SC	0.217136	0.541635	<0.001	Yes
SC_YN	YN	0.443639	0.5913945	<0.001	Yes

Note: p-values from one-sided Wilcoxon rank-sum test (alternative: median candidate < median control). The statistical significance level was set at $p<0.05$.
