# Supplementary material for: Whole‐Genome Resequencing Reveals the Genetic Diversity and Adaptive Evolution of Rhabdophis nuchalis
Source: Ecol Evol. 2026 Sep 28;16(10):e74438. doi: 10.1002/ece3.74438 (PMC13619356; doi:10.1002/ece3.74438)
Supplement: Supplementary file 3 [file ECE3-16-e74438-s003.pdf]

Supplementary Table 4. Number of Windows and Genes Identified by Selective Sweep Analysis in the Population

Population Pair	Number of Candidate Windows	Number of Genes
HB-SC	5598	1315
HB-SX	2145	498
HB-YN	5999	1034
SC-SX	5501	1104
SC-YN	5423	1143
SX-YN	5829	923
