# Supplementary material for: Whole‐Genome Resequencing Reveals the Genetic Diversity and Adaptive Evolution of Rhabdophis nuchalis
Source: Ecol Evol. 2026 Sep 28;16(10):e74438. doi: 10.1002/ece3.74438 (PMC13619356; doi:10.1002/ece3.74438)
Supplement: Supplementary file 4 [file ECE3-16-e74438-s004.pdf]

Supplementary Table 2. Sample Data Information Sheet

Sample ID	GroupCode	Mean Coverage	% Mapped	Q20	Q30
ZGX410	HB1	10.7X	99.80%	98.64%	96.18%
ZGX411	HB2	10.0X	99.80%	98.72%	96.43%
ZGX412	HB3	11.3X	99.80%	98.63%	96.15%
ZGX564	SX1	12.6X	99.80%	98.85%	96.79%
ZGX565	SX2	10.4X	99.80%	98.72%	96.44%
ZGX566	SX3	14.4X	99.80%	98.81%	96.74%
ZGX578	SX4	11.4X	99.80%	98.81%	96.73%
ZGX579	SX5	11.7X	99.80%	98.73%	96.47%
ZGX580	SX6	9.7X	99.70%	98.70%	96.37%
ZGX581	SX7	10.7X	99.80%	98.63%	96.18%
ZGX582	SX8	9.7X	99.80%	98.78%	96.63%
ZGX585	SC1	11.9X	99.60%	98.79%	96.66%
ZGX586	SC2	13.1X	99.60%	98.80%	96.67%
ZGX587	SC3	14.4X	99.60%	98.80%	96.68%
ZGX588	SC4	11.1X	99.60%	98.69%	96.35%
ZGX589	SC5	11.0X	99.60%	98.73%	96.46%
ZGX592	YN1	11.9X	99.60%	98.77%	96.59%
ZGX593	YN2	12.7X	99.60%	98.80%	96.68%
ZGX594	YN3	10.6X	99.60%	98.76%	96.56%
**Mean**		**11.5X**	**99.71%**	**98.75%**	**96.51%**
