# Supplementary material for: Whole‐Genome Resequencing Reveals the Genetic Diversity and Adaptive Evolution of Rhabdophis nuchalis
Source: Ecol Evol. 2026 Sep 28;16(10):e74438. doi: 10.1002/ece3.74438 (PMC13619356; doi:10.1002/ece3.74438)
Supplement: Supplementary file 5 [file ECE3-16-e74438-s005.pdf]

Supplementary Table 1. *Rhabdophis nuchalis* sample collection information

No.	Sample ID	Sampling Location	GroupCode
1	ZGX410	Shennongjia, Hubei	HB1
2	ZGX411	Shennongjia, Hubei	HB2
3	ZGX412	Shennongjia, Hubei	HB3
4	ZGX564	Ankang, Shaanxi	SX1
5	ZGX565	Ankang, Shaanxi	SX2
6	ZGX566	Ankang, Shaanxi	SX3
7	ZGX578	Ankang, Shaanxi	SX4
8	ZGX579	Ankang, Shaanxi	SX5
9	ZGX580	Ankang, Shaanxi	SX6
10	ZGX581	Ankang, Shaanxi	SX7
11	ZGX582	Ankang, Shaanxi	SX8
12	ZGX585	Panzhihua, Sichuan	SC1
13	ZGX586	Panzhihua, Sichuan	SC2
14	ZGX587	Panzhihua, Sichuan	SC3
15	ZGX588	Panzhihua, Sichuan	SC4
16	ZGX589	Panzhihua, Sichuan	SC5
17	ZGX592	Dali Bai Autonomous Prefecture, Yunnan	YN1
18	ZGX593	Dali Bai Autonomous Prefecture, Yunnan	YN2
19	ZGX594	Dali Bai Autonomous Prefecture, Yunnan	YN3
